# Rainstorm-induced organic matter pulses: A key driver of carbon emissions from inland waters

**DOI:** 10.1016/j.xinn.2024.100746

**Published:** 2025-01-04

**Authors:** Lei Zhou, Yongqiang Zhou, Yunlin Zhang, Erik Jeppesen, Gesa A. Weyhenmeyer

**Affiliations:** 1 State Key Laboratory of Soil and Sustainable Agriculture, Institute of Soil Science, Chinese Academy of Sciences, Nanjing 211135, China; 2 Taihu Laboratory for Lake Ecosystem Research, State Key Laboratory of Lake Science and Environment, Nanjing Institute of Geography and Limnology, Chinese Academy of Sciences, Nanjing 211135, China; 3 Key Laboratory of Lake and Watershed Science for Water Security, Nanjing Institute of Geography and Limnology, Chinese Academy of Sciences, Nanjing 211135, China; 4 University of Chinese Academy of Sciences, Beijing 100049, China; 5 Department of Ecoscience and Centre for Water Technology (WATEC), Aarhus University, Aarhus 8000, Denmark; 6 Sino-Danish Centre for Education and Research, Beijing 100190, China; 7 Limnology Laboratory, Department of Biological Sciences and Centre for Ecosystem Research and Implementation, Middle East Technical University, Ankara 06800, Turkey; 8 Institute for Ecological Research and Pollution Control of Plateau Lakes, School of Ecology and Environmental Science, Yunnan University, Kunming 650091, China; 9 Department of Ecology and Genetics/Limnology, Uppsala University, Uppsala 75236, Sweden

## Abstract

Numerous rivers and lakes in the monsoon climate zone are heavily influenced by frequent rainstorms that mobilize dissolved organic matter (DOM) from pristine or urbanized environments into downstream lakes. Of particular concern is the mobilization of DOM from anthropogenic effluents, which are commonly enriched in aliphatic compounds that can be easily degraded by microorganisms. Rapid degradation of highly biodegradable DOM, in turn, may cause significant depletion of dissolved oxygen in the water, which, by creating anoxic conditions at the bottom water-sediment interface, promotes microbial production of CO_2_ and CH_4_. Further investigations based on high-frequency monitoring and novel techniques such as ultra-high-resolution mass spectrometry and isotopic measurements, are needed to elucidate the processes and mechanisms by which pulsed aliphatic inputs impact lake carbon emissions.

## Background and importance

**Figure 1:**
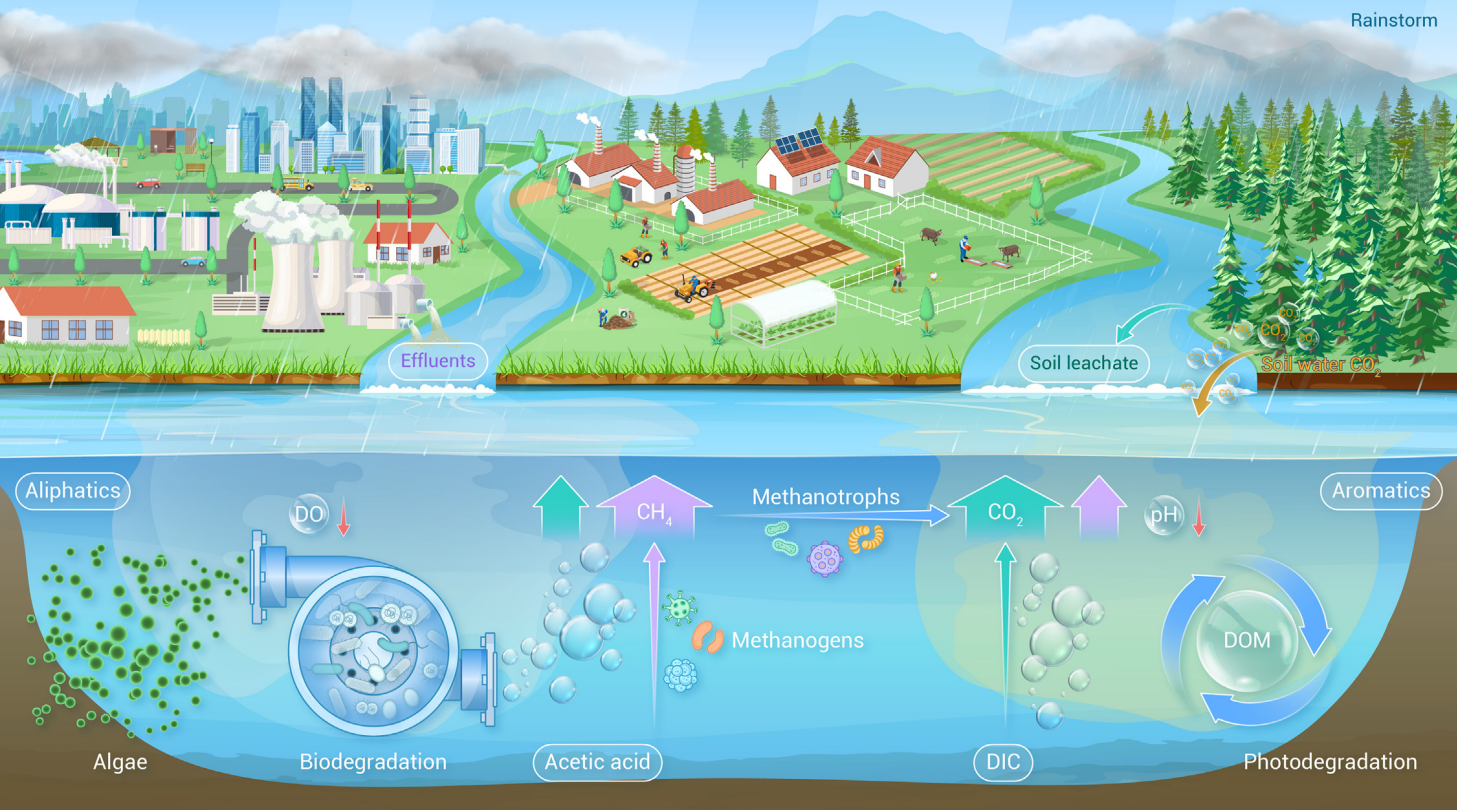
Rainstorm-induced organic matter inputs drive carbon emissions from inland waters Shown is a schematic of how a rainstorm event increases input of terrestrial dissolved organic matter (DOM), alters water chemistry, and promotes the outgassing of carbon dioxide (CO_2_) and methane (CH_4_) in downstream lake ecosystems, particularly in inflow areas. DO, dissolved oxygen; DIC, dissolved inorganic carbon.

Lakes and other inland waters are hotspots for transforming terrestrial organic matter as they receive, actively process, and transport up to 5.1 petagrams of carbon (PgC) annually^1^ despite covering less than 3.7% of the Earth’s non-glaciated land surface. Global estimates of lake carbon cycling have been revised several times, but recent ones suggest that carbon dioxide (CO_2_) fluxes from lakes and other inland waters to the atmosphere can reach up to 3.9 PgC year^−1^,^1^ while lake methane (CH_4_) emissions contribute about 0.4 Pg CH_4_ year^−1^, accounting for nearly half of the annual global CH_4_ emissions.^2^ Carbon emissions result from the transformation of organic matter in terrestrial and aquatic ecosystems. In aquatic ecosystems, organic matter mainly consists of dissolved organic carbon (DOC), which can constitute up to 90% of the total organic carbon pool in rivers and lakes, and it plays a crucial role in the carbon cycle of inland waters.^3^ The degradability of lake organic matter is largely determined by its source and chemical composition. Terrestrial organic matter, usually the primary contributor to the lake organic carbon pool, undergoes significant microbial and photochemical degradation after entering lakes (Figure 1). Freshwater lakes are typically in a state of CO_2_ and CH_4_ supersaturation, and mesocosm and laboratory experiments have shown that adding fresh DOC and organic matter may significantly increase CO_2_ and CH_4_ production,^4^ emphasizing that investigating the biogeochemical cycling of terrestrial organic matter is crucial for understanding the dynamics of lake carbon emissions.

The decomposition of organic matter through biogeochemical processing produces biogenic solutes, including DOC and various forms of nitrogen. Their dynamics in lakes are significantly modulated by hydrological processes, especially during rainstorm events (precipitation ≥ 50 mm day^−1^). Such events cause fast inundation of the water table, moving from an organic-poor to an organic-rich layer. River water primarily comes from headwaters that drain rapidly over shallow, permeable, wet soils in well-connected landscapes, integrating soil biogeochemical signals along the shallow flow paths enriched in DOC.^5^ Flood-derived concentrations of particulate organic carbon are much lower than those of DOC and vary inversely with discharge in pristine rivers.^3^ Rainstorms and floods typically mobilize large amounts of dissolved organic matter (DOM) from soil leaching, vegetation decay, and agricultural and residential effluents into downstream rivers and lakes (Figure 1).^6^ Biogenic DOC concentrations typically increase with increasing surface water discharge (flushing), and their loads often rise with discharge regardless of concentration-discharge relationships, as discharge typically increases by orders of magnitude during transitions from dry to rainstorm conditions.^5^ For instance, in the eastern forests of the United States, DOC mobilized during rainfall events accounted for 86% of the annual export, with rainstorms contributing 57% of the annual DOC yield despite occurring only 4.8% of the year (approximately 18 days).^7^ In Fish Creek catchment, Alaska, the onset of rainstorms resulted in an almost 30-fold increase in streamflow and a sharp rise in DOC concentrations from about 2 mg L^−1^ before the event to about 9 mg L^−1^ afterward.^8^ Similarly, following hurricane Irene in New York state, Esopus Creek experienced a staggering 330-fold increase in flow rates, with DOC concentrations reaching more than twice the usual levels.^9^ Remarkably, within the 5-day rainstorm event, this single stream contributed 43% of the total annual DOC export.^9^ These large increases in DOC concentrations and loads may be closely associated with sanitary sewer overflows and increased agricultural runoff during rainstorms.^10^ By contrast, very high flows can result in low concentrations of DOC and other dissolved pollutants in rivers and some lakes due to dilution, as supported by a study of Lake Qiandao in China, where a rainstorm resulted in a sharp decrease in DOC, from about 2.0 mg L^−1^ to about 1.0 mg L^−1^. Thus, increased mobilization and dilution during rainstorms and floods may have opposite impacts on DOC concentrations.^10^ These results highlight the need for frequent and high-resolution monitoring to accurately capture the transient yet influential nature of rainstorm events on lake carbon cycling and greenhouse gas emissions.

## Challenges

Although rainstorms trigger increases in riverine DOC concentrations and fluxes to lakes, the understanding of how DOM quality, microbial activity, and carbon emissions, along with their interactions, respond to such extreme events remains limited.^6^ Carbon emissions from lakes during rainstorms are strongly dependent on the sources of DOM. Hydrologic and biogeochemical processes in pristine landscapes may differ significantly from those in intensively developed and urbanized catchments (Figure 1). Impervious landscapes facilitate surface runoff, while sewer and stormwater pipes enhance rapid subsurface flow, resulting in shallow hydrologic flow paths.^5^ In pristine environments, terrestrial DOM from soil leachate is characterized by a higher proportion of aromatic and carboxylated lipid ring compounds, which is associated with an increased propensity for photochemical reactions. Photochemical degradation breaks down highly oxidized and aromatic DOM, continuously forming low-molecular-weight compounds that serve as substrates for carbon emissions, including CO_2_ and CH_4_ (Figure 1). Photodegradation of soil DOC can directly produce CO_2_, and the rapid utilization of unstable photoproducts by microbial metabolism can also lead to production of CO_2_ and CH_4_. In comparison, DOM from anthropogenic wastewater in densely urbanized regions is enriched in aliphatic components as well as nitrogen- and sulfur-enriched (CHON, CHOS) assemblages (Figure 1). These are readily degraded by microorganisms, with biodegradable DOC levels as high as 70%. Rapid degradation of highly biodegradable DOM from municipal wastewater can significantly deplete dissolved oxygen in the water, creating anoxic conditions at the bottom water-sediment interface, which further promotes microbial CO_2_ and CH_4_ production (Figure 1).^6^ Some studies have shown that the contribution of CH_4_ production from acetate degradation can be substantially enhanced by DOM reactivity.^6^ In recent years, microbiomics studies in anaerobic environments have shown that certain archaea can directly utilize macromolecular organic matter to produce CH_4_. Additionally, CH_4_ supersaturation has been found in aerobic lake waters.^6^ These findings broaden the understanding of methanogenic pathways in lakes and the role of DOM composition in CH_4_ emissions. However, how the intricate mechanisms by which DOM activity and degradation by-products, such as acetic acid, that serve as CH_4_ precursors shape the structure of microbial communities involved in CH_4_ metabolism and CO_2_ and CH_4_ emissions during rainstorms remains unclear. Overall, rainstorms significantly influence carbon emissions. High flows during these events mobilize large amounts of DOC from the catchment, increasing the mobilization of soil- or wetland-derived CO_2_^3^ and CH_4_.^6^ This creates inflow zone areas where DOC concentrations are high, acting as hotspots for CO_2_ and CH_4_ emissions. The mean CO_2_ flux from a large reservoir increased significantly from −3.8 mmol m^−2^ day^−1^ to 13.2 mmol m^−2^ day^−1^ and mean CH_4_ efflux from 0.06 mmol m^−2^ day^−1^ to 0.12 mmol m^−2^ day^−1^ after rainstorms.^6^ Terrestrial soil DOM is rich in organic acids and can facilitate CO_2_ escape by lowering the reservoir’s pH from 9.8 to 7.5, dissolved oxygen (DO) from 9.5 mg L^−1^ to 8.3 mg L^−1^, and Chl-*a* from 30 μg L^−1^ to 1 μg L^−1^ during the pre- to post-rainstorm period.^6^ The rapid decrease in pH converts dissolved inorganic carbon from carbonate (CO_3_^2−^) and bicarbonate (HCO_3_^−^) to free CO_2_ (Figure 1).^4^ Consequently, CO_2_ and CH_4_ emissions during rainstorms are markedly higher than during normal- and low-flow periods.^3^^,^^6^ However, quantifying the relative importance of riverine CO_2_ and CH_4_ inputs versus in-lake transformation of terrestrial DOM to lake carbon emissions during rainstorms remains challenging.

## Opportunities

Advanced and innovative analytical techniques are essential to unravel the complex processes between DOM quality, microbial activity, and carbon emissions. Identifying DOM quality with novel methods is crucial for determining its fate in the lake environment. Different DOM sources have unique compositions, and identifying specific compounds within a sample can trace their origins. Isotopic ratios of δ^13^C-DOC and radiocarbon Δ^14^C-DOC, along with ultra-high-resolution mass spectrometry, enable detailed characterization of DOM sources and molecular composition. The stable isotope signatures δ^13^C-CO_2_ and δ^13^C-CH_4_ provide valuable insights into CO_2_ and CH_4_ sources. Methanogens are classified into methylotrophs, aceticlotrophs, and hydrogenotrophs based on metabolic pathways. The fractionation coefficient α_C_, calculated as (δ^13^C-CO_2_ + 1,000)/(δ^13^C-CH_4_ + 1,000), is an important indicator of isotopic fractionation patterns, allowing differentiation of the metabolic pathways used by methanogens. Methanogenic processes driven by hydrogenotrophic methanogens typically deplete δ^13^C-CH_4_ and elevate α_C_ values (>1.055), whereas those dominated by acetotrophic methanogens show enriched δ^13^C-CH_4_ and lower α_C_ values (<1.055).^6^ Metagenomic technologies together with qPCR based on functional genes (e.g., *mcrA* or *pmoA*) can elucidate variations in the abundance, structure, and function of the associated microbial communities.

## Conclusion and future work

In summary, during rainstorms, terrestrial DOM can be rapidly mobilized and degraded, leading to significant decreases in pH and dissolved oxygen levels and high production and emission of CO_2_ and CH_4_ from downstream-linked lakes (Figure 1). However, our understanding of how rainstorms alter the composition and fate of DOM, and the subsequent effects on dissolved inorganic carbon, CO_2_, and CH_4_ gas fluxes and sources, is limited. Therefore, continuous and high-frequency monitoring of DOM quality, acetic acid, methanogen abundance, and carbon emissions is important to elucidate the processes and mechanisms behind the impact of pulse-like DOM inputs on lake carbon emissions during rainstorms. There is a need to advance technologies (e.g., ultra-high-resolution mass spectrometry and isotopic measurements) along with data-driven (deep learning) and process-based models to monitor and make predictions for biogeochemical hotspots experiencing accelerated or slower carbon cycling under rainstorms and floods. Existing carbon cycling monitoring data are highly fragmented, complicating the characterization of long- and short-term biogeochemical responses under rainstorms. Compiling local carbon cycling data into large global datasets and using deep learning models and techniques to expand *in situ* biogeochemical monitoring campaigns would be beneficial.^5^^,^^10^ Deep learning models, together with *in situ* and satellite observations, are promising tools for tracking the high-frequency dynamics of carbon cycling during rainstorms and floods in inland waters.

## Acknowledgments

This work was supported by the 10.13039/501100001809National Natural Science Foundation of China (42322104, 42471123, and 42207447); the 10.13039/501100004739Youth Innovation Promotion Association, CAS (2021312); the Provincial Natural Science Foundation of Jiangsu (BK20220162); and the Ecological Environment Research Project of Jiangsu Province of China (2023003). E.J. was supported by the TÜBITAK program BIDEB2232 (project 118C250), and G.A.W. received financial support from the 10.13039/501100004359Swedish Research Council (VR grant 2020-03222 and 10.13039/501100001862Formas grant 2020-01091). We thank Anne Mette Poulsen and Xifan Gao for valuable additions. The funders had no role in study design, data collection and analysis, decision to publish, or preparation of the manuscript.

## Author contributions

Y. Zhou conceptualized and designed the study. L.Z. drafted the initial manuscript, and Y. Zhang, E.J., and G.A.W. critically reviewed and revised the text. All authors contributed to and approved the manuscript.

## Declaration of interests

E.J. is a Steering Committee member of *The Innovation* and was blinded from reviewing or making final decisions on the manuscript. Peer review was handled independent of this member and their research group.
